# Metabolic engineering for efficient supply of acetyl-CoA from different carbon sources in *Escherichia coli*

**DOI:** 10.1186/s12934-019-1177-y

**Published:** 2019-08-06

**Authors:** Shasha Zhang, Wei Yang, Hao Chen, Bo Liu, Baixue Lin, Yong Tao

**Affiliations:** 1 Chinese Academy of Sciences Key Laboratory of Microbial Physiological and Metabolic Engineering, Institute of Microbiology, Chinese Academy of Sciences, Beijing, 100101 China; 2 University of Chinese Academy of Sciences, Beijing, 100049 China

**Keywords:** *N*-Acetylglutamate, Acetyl-CoA, Glucose, Acetate, Fatty acid

## Abstract

**Background:**

Acetyl-CoA is an important metabolic intermediate and serves as an acetylation precursor for the biosynthesis of various value-added acetyl-chemicals. Acetyl-CoA can be produced from glucose, acetate, or fatty acids via metabolic pathways in *Escherichia coli*. Although glucose is an efficient carbon source for acetyl-CoA production, the pathway from acetate to acetyl-CoA is the shortest and fatty acids can produce acetyl-CoA through fatty acid oxidation along with abundant NADH and FADH_2_. In this study, metabolically engineered *E. coli* strains for efficiently supplying acetyl-CoA from glucose, acetate, and fatty acid were constructed and applied in one-step biosynthesis of *N*-acetylglutamate (NAG) from glutamate and acetyl-CoA.

**Results:**

A metabolically engineered *E. coli* strain for NAG production was constructed by overexpressing *N*-acetylglutamate synthase from *Kitasatospora setae* in *E. coli* BW25113 with *argB* and *argA* knockout. The strain was further engineered to utilize glucose, acetate, and fatty acid to produce acetyl-CoA. When glucose was used as a carbon source, the combined mutants of ∆*ptsG::glk*, ∆*galR::zglf*, ∆*poxB::acs*, ∆*ldhA*, and ∆*pta* were more efficient for supplying acetyl-CoA. The acetyl-CoA synthetase (ACS) pathway and acetate kinase-phosphate acetyltransferase (ACK-PTA) pathway from acetate to acetyl-CoA were investigated, and the ACK-PTA pathway showed to be more efficient for supplying acetyl-CoA. When fatty acid was used as a carbon source, acetyl-CoA supply was improved by deletion of *fadR* and constitutive expression of *fadD* under the strong promoter CPA1. Comparison of acetyl-CoA supply from glucose, acetate and palmitic acid revealed that a higher conversion rate of glutamate (98.2%) and productivity (an average of 6.25 mmol/L/h) were obtained when using glucose as a carbon source. The results also demonstrated the great potential of acetate and fatty acid to supply acetyl-CoA, as the molar conversion rate of glutamate was more than 80%.

**Conclusions:**

Metabolically engineered *E. coli* strains were developed for NAG production. The metabolic pathways of acetyl-CoA from glucose, acetate, or fatty acid were optimized for efficient acetyl-CoA supply to enhance NAG production. The metabolic strategies for efficient acetyl-CoA supply used in this study can be exploited for other chemicals that use acetyl-CoA as a precursor or when acetylation is involved.

**Electronic supplementary material:**

The online version of this article (10.1186/s12934-019-1177-y) contains supplementary material, which is available to authorized users.

## Background

Acetyl-CoA, a metabolite in primary carbon metabolism, is involved in various biological processes. It can act as an intermediate to transfer an acetyl group during the biosynthesis of diverse acetyl-chemicals [1]. Acetyl-CoA is also a platform chemical for producing various high-value products, such as isoprenoids (used as flavors, biofuels, pharmaceuticals, and vitamins) [2], 1-butanol [3], 3-hydroxypropionate (one of the 12 biology-based chemical products with the most potential according to the Department of Energy, USA) [4], and polyhydroxyalkanoates [5]. Metabolic engineering targeting the intracellular acetyl-CoA pool is typically conducted to enhance the acetyl-CoA supply for producing high-value added chemicals using acetyl-CoA as a precursor [6, 7].

**Fig. 1:**
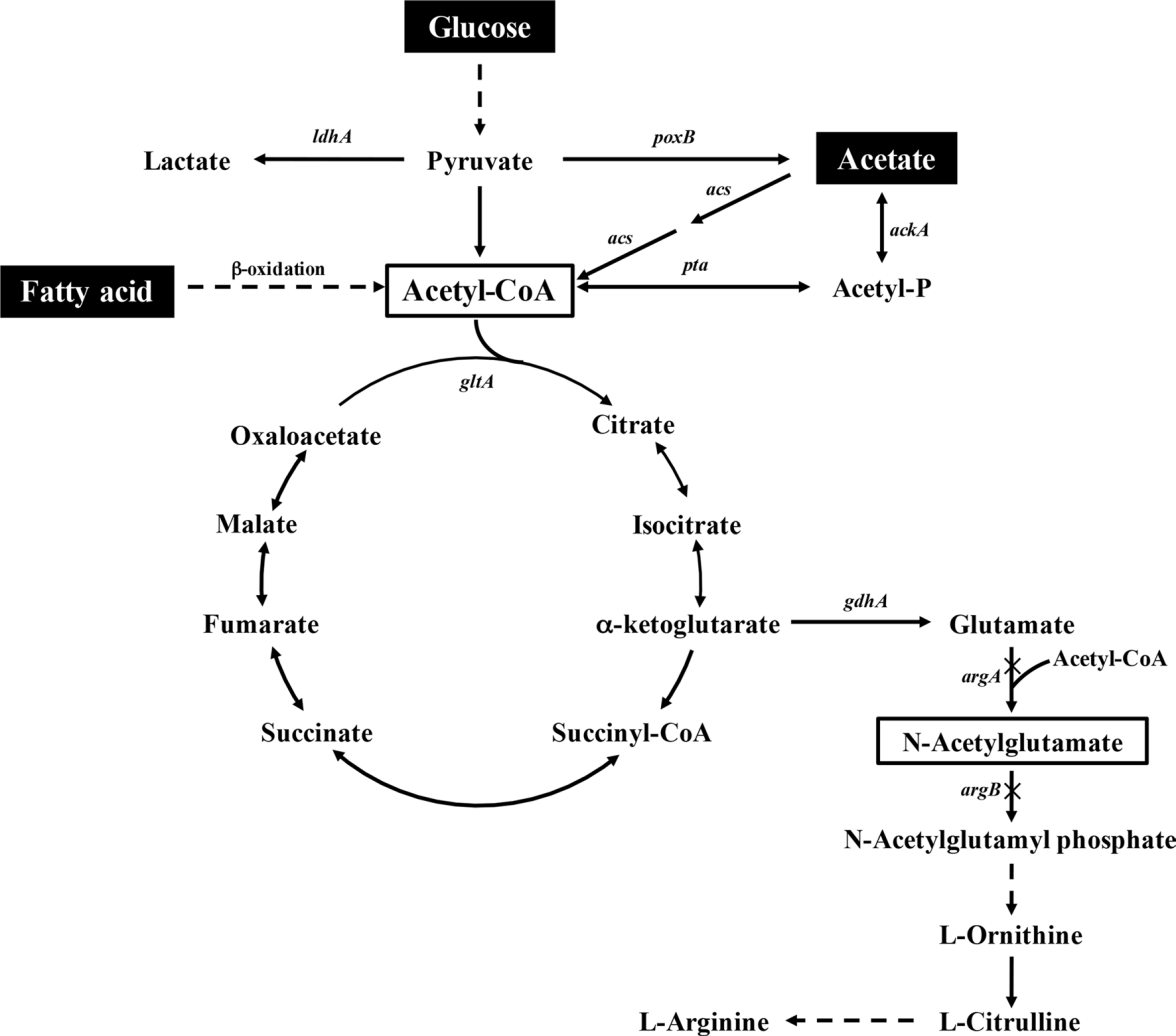
Overview of acetyl-CoA metabolism in *Escherichia coli* and the biosynthesis pathway of NAG

Acetyl-CoA can be synthesized from glucose, acetate, and fatty acid in *Escherichia coli* (Fig. 1). Glucose is the most commonly used carbon source in *E. coli*, which produces acetyl-CoA via an efficient glycolysis pathway. Glucose is first converted to pyruvate through the glycolytic pathway, and then into acetyl-CoA by pyruvate dehydrogenase under aerobic conditions or by pyruvate-formate lyase under anaerobic conditions with the release of CO_2_. The loss of carbon in this step is associated with a decrease in the theoretical carbon recovery rate of 66.7% when using glucose as an acetyl-CoA source (Table 1). In recent studies, Mainguet et al. [8] and Bogorad et al. [9] had reported that 3 mol acetyl-CoA could be produced from 1 mol glucose by a reconstructed glyoxylate shunt or a non-oxidative glycolysis. The studies are still in the stage of proof of concept and more works are needed to make it possible for industrial application. Recently, acetate was used as an alternative carbon source and to produce chemicals such as succinate [10, 11], itaconic acid [12], isobutanol [13], and polyhydroxyalkanoates [14]. Two pathways can convert acetate to acetyl-CoA: (1) the reversible acetate kinase-phosphate acetyltransferase (ACK-PTA) pathway and (2) the irreversible ACS pathway catalyzed by acetyl-CoA synthetase (ACS). ACS is a high-affinity enzyme with a low *K*_*m*_ of 200 μM for acetate [15], while the ACK-PTA pathway can only act at a relatively high concentration, as the *K*_*m*_ values of both enzymes for their substrates are 7–10 mM [16]. More ATP is consumed in the ACS pathway when AMP is formed compared to the ACK-PTA pathway in which ADP is formed [17, 18]. Fatty acids can also be used to produce acetyl-CoA along with abundant NADH and FADH_2_ via fatty acid oxidation [19]. For example, oxidation of 1 mol palmitic acid (a type of fatty acid) can produce 7 mol NADH and 7 mol FADH_2_. The used of fatty acids is very efficient and can result in product yields that are significantly higher than those obtained from other carbon sources because of its high atom economy [20]. The theoretical carbon recovery rate of acetate and fatty acid as an acetyl-CoA source is 100%, which is higher than that of glucose.

**Table 1:** The equation of different carbon sources producing acetyl-CoA

Carbon source	Equation	Carbon recovery rate
Glucose	C_6_H_12_O_6_ → 2 Acetyl-CoA + 2 CO_2_ + 4 NADH + 2 ATP	2/3
Acetate	C_2_H_4_O_2_ + ATP → Acetyl-CoA	1
Palmitic acid	C_16_H_32_O_2_ + 2 ATP → 8 Acetyl-CoA + 7 NADH + 7 FADH_2_	1

NAG is the initial precursor of arginine in the arginine biosynthetic pathway in microorganisms and plants and is essential for the biosynthesis of l-citrulline. In addition, NAG is an essential allosteric cofactor of mitochondrial carbamyl phosphate synthetase I, the first and rate-limiting enzyme of the urea cycle in ureotelic animals. Studies of NAG synthesis are important for improving the biosynthesis of l-citrulline, l-arginine, and other derivatives. Acetyl-CoA is used to provide an acetyl group in this one-step reaction of NAG synthesis from glutamate.

In this study, a metabolically engineered *E. coli* was developed for NAG production by introducing genes that catalyze NAG formation into the modified host strain. We focused on metabolic engineering for efficiently supplying acetyl-CoA from glucose, acetate, and fatty acid (particularly, palmitic acid). The results provide insight into methods for efficiently supplying acetyl-CoA for chemical synthesis using acetyl-CoA as a precursor or requiring acetylation. Additionally, metabolic engineering for NAG synthesis provides a foundation for industrial production of NAG and biosynthesis of arginine and its derivatives.

## Results

### Construction of biosynthetic pathway of NAG in *E. coli*

**Fig. 2:**
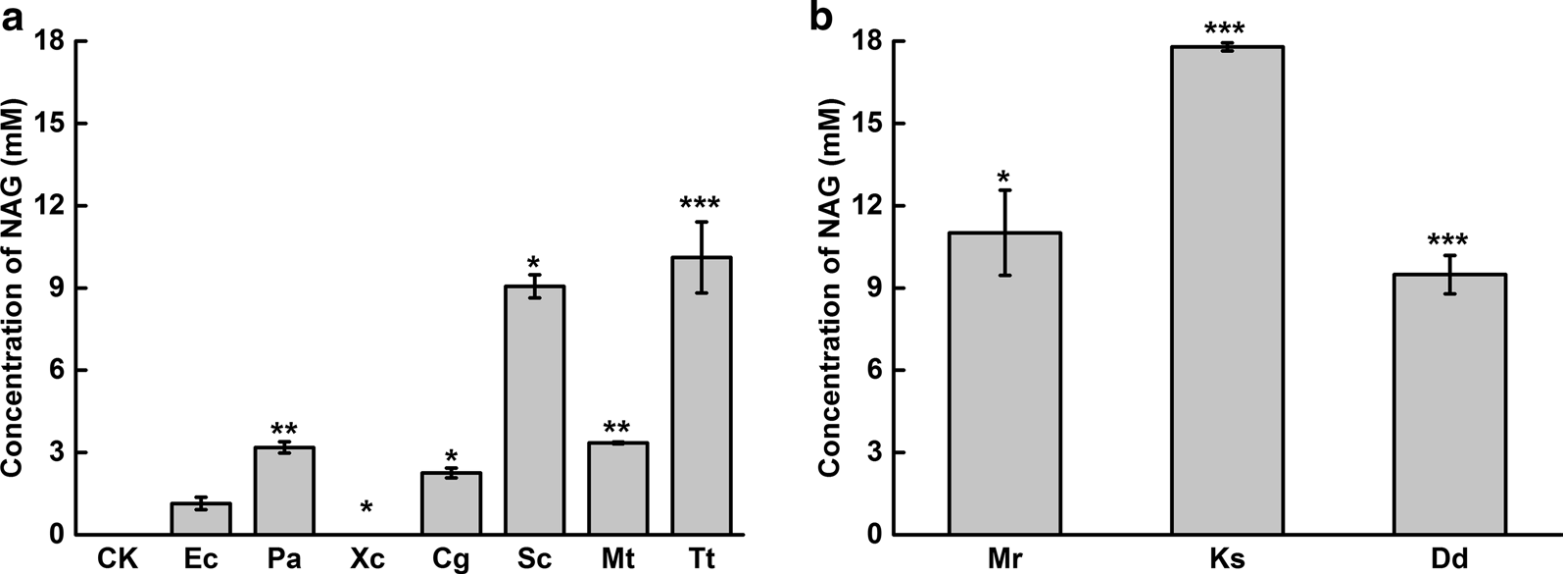
Effects of *N*-acetylglutamate synthase (NAGS) from different species on NAG production. **a** Effects of NAGS reported in references on NAG production. **b** Effects of NAGS not previously reported on NAG production. Ec = *E. coli*, Pa = *Pseudomonas aeruginosa*, Xc = *Xanthomonas campestris*, Cg = *Corynebacterium glutamicum*, Sc = *Streptomyces coelicolor*, Mt = *Mycobacterium tuberculosis*, Tt = *Thermus thermophilus*, Mr = *Meiothermus ruber*, Ks = *Kitasatospora setae*, Dd = *Deinococcus deserti*

In *E. coli*, NAG was the precursor for arginine synthesis. *N*-acetylglutamate kinase encoded by *argB* and bi-functional *N*-acetylglutamate synthase encoded by *argA* were involved in the arginine biosynthesis pathway (Fig. 1). For NAG accumulation, *argB* and *argA* in *E. coli* BW25113 were knocked out resulted in strain N00 which was used as chassis for NAG production. *N*-Acetylglutamate synthase (NAGS) with high catalytic efficiency was then screened for efficient synthesis of NAG. The reported NAGS genes from *E. coli*, *Pseudomonas aeruginosa*, *Xanthomonas campestris*, *Corynebacterium glutamicum*, *Streptomyces coelicolor*, *Mycobacterium tuberculosis*, and *Thermus thermophiles* [21–27] were overexpressed in *E. coli* under the control of the pBAD promoter in plasmids pN01, pN02, pN03, pN05, pN06, pN08, and pN14, respectively. The plasmid was transformed into the host strain N00. The strains were cultured and used for whole-cell biocatalysis of NAG. After 8 h of whole-cell bioconversion, 10.12 mM NAG was obtained from 50 mM sodium glutamate and 50 mM glucose by the strain with NAGS from *T. thermophiles* which was higher than that from other NAGSs (Fig. 2a). However, the protein expression of Tt-NAGS (NAGS from *T. thermophiles*) was unstable. Although the strategies of codon-optimization, co-expression with chaperone, and lower temperature (data not shown) had been tried, no significant improvement on Tt-NAGS expression was observed. Therefore, we search for new NAGS in the NCBI database based on the reported amino acid sequences of the NAGS from *S. coelicolor* and *T. thermophiles*. NAGSs from *Meiothermus ruber*, *Kitasatospora setae*, and *Deinococcus deserti* were selected and cloned in host N00. A higher amount of NAG (17.89 mM) was obtained by the strain 0019 with Ks-NAGS. Enzyme measurements of Ec-NAGS, Tt-NAGS and Ks-NAGS from *E. coli*, *T. thermophiles* and *K. setae* were performed using purified recombinant proteins. The enzymatic analysis of the specific activity of Ec-NAGS, Tt-NAGS and Ks-NAGS showed that Ks-NAGS from *K. setae* had higher specific activity than the other two (Table 2). Thus, strain 0019 was used thereafter for metabolic engineering of acetyl-CoA supply from different carbon sources.

**Table 2:** The specific activity of Ec-NAGS, Tt-NAGS and Ks-NAGS from *E. coli*, *T. thermophiles* and *K. setae* respectively

Enzyme	Specific activity (μmol/min/mg)
Ec-NAGS	3.7 ± 0.1
Tt-NAGS	5.4 ± 0.4
Ks-NAGS	8.0 ± 0.1

### Metabolic engineering for efficient supply of acetyl-CoA from glucose

**Fig. 3:**
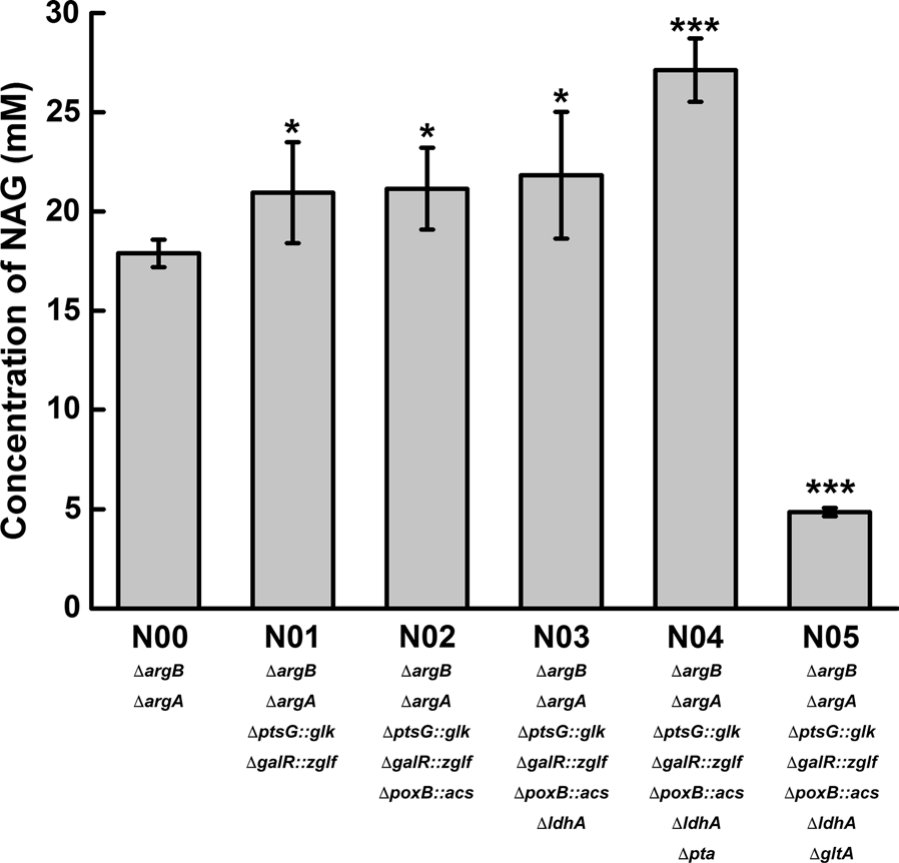
Production of NAG by engineered strain from glutamate and glucose. Engineered host strains transformed with plasmid pNAG19 were induced and suspended in a reaction mixture containing 50 mM sodium glutamate and 50 mM glucose. The bioconversion reactions were performed at 37 °C and 200 rpm for 8 h

When using glucose as a carbon source to produce acetyl-CoA, the glucose utilization system was first modified by replacing native *ptsG* and *galR* with *glk* and *zglf* (galactose: H^+^ symporter from *Zymomonas mobilis*) in the chromosome of host N00 based on a previous study which showed that PTS-^–^ strains recruiting *Z. mobilis* glucose facilitator Glf and *E. coli* Glk had higher glucose utilization rates than wild-type strain [28]. The resulting host N01 (BW25113, ∆*argB,* ∆*argA*, ∆*ptsG::glk,* ∆*galR::zglf*) was then transformed with plasmid pN19. The recombinant strain 0119 exhibited increased NAG production to 20.95 mM after 8 h of bioconversion (Fig. 3, shown as N01).

Because acetyl-CoA was converted via pyruvate when using glucose as a carbon source, the bypass pathways of pyruvate were then blocked in host strain N01 to eliminate by-products and increase the pyruvate pool. The host strain N02 was obtained by replacing the pyruvate oxidase gene, *poxB*, with the acetyl-CoA synthetase gene, *acs*. The lactate dehydrogenase gene, *ldhA*, was then inactivated in N02 to produce the host N03. As shown in Fig. 3, this manipulation slightly increased NAG production.

*pta* and *gltA*, which react in the bypass pathways of acetyl-CoA, were inactivated in host N03 to produce hosts N04 and N05, respectively. When using recombinant strain 0419 as a whole-call biocatalyst, NAG production was clearly improved. In contrast, deletion of *gltA* greatly reduced NAG production (Fig. 3, shown as N05). Based on these results, modification of the glucose utilization system and disruption of the bypass pathways of pyruvate and acetyl-CoA increased the acetyl-CoA pool. This led to a 51.6% increase in NAG production to 27.12 mM.

### Metabolic engineering of the strain for efficient supply of acetyl-CoA from acetate

**Fig. 4:**
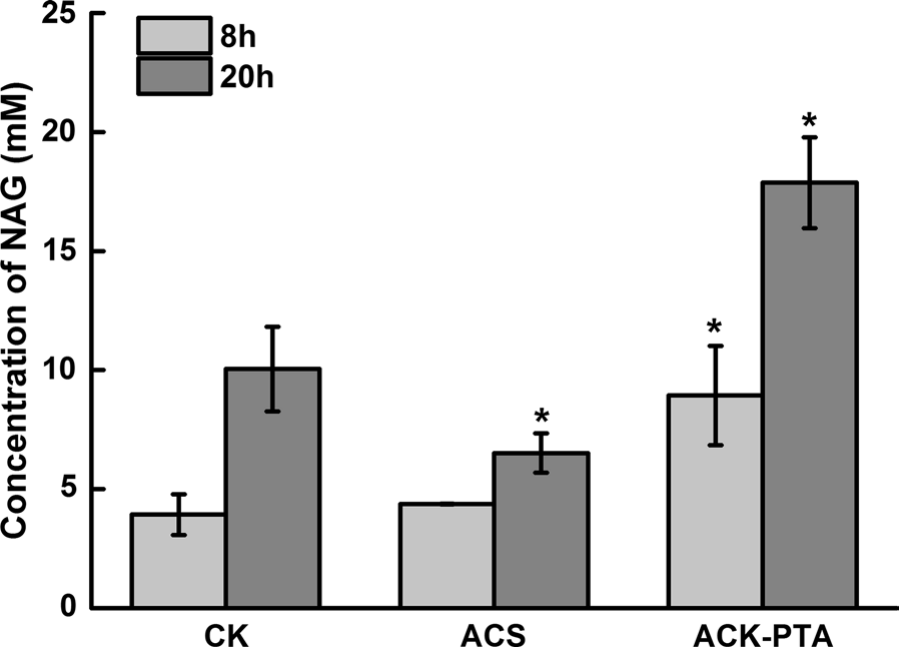
Production of NAG by engineered strain from glutamate and acetate. The bioconversion medium used here contained 50 mM sodium glutamate and 100 mM sodium acetate. The bioconversion reactions were performed at 37 °C and 200 rpm. H_2_SO_4_ was added at different reaction times to adjust the pH value

There are two pathways in *E. coli* that can convert acetate to acetyl-CoA: (1) ACS pathway can function at a low concentration of acetate but consumes more ATP. (2) ACK-PTA pathway requires less ATP but can only function at a relatively high concentration of acetate. These two pathways were investigated in our study by overexpressing *acs* or *ackA*-*pta* in *E. coli*. The plasmids overexpressing ACS (pAcs) or AckA-Pta (pAck) were co-transformed with pN19 into the host strain N00 to form the recombinant strains 0019Acs and 0019Ack, respectively. In the whole-cell bioconversion acetate was used as substrate for acetyl-CoA synthesis and low concentration of glucose (2 mM) was added to the system for initial supply of ATP. As shown in Fig. 4, after 20 h of bioconversion from 50 mM sodium glutamate and 100 mM sodium acetate, 6.52 mM NAG was produced by strain 0019Acs overexpressing the ACS pathway, while 17.88 mM NAG was produced by strain 0019Ack overexpressing the ACK-PTA pathway. Strain 0019Acs produced even less NAG than the control strain 0019, which produced 10.05 mM NAG after 20 h of bioconversion. Additionally, after overnight induction, the biomass of strain 0019Acs was 40% lower than those of the other two strains (data not shown). This indicated that the overexpression of *acs* might affect the normal metabolism in this strain.

The above results demonstrated that overexpression of the *ackA*-*pta* operon in a plasmid can enhance the pathway that converts acetate to acetyl-CoA, leading to a 77.9% increase in the acetyl-CoA supply for NAG production when using acetate as a carbon source.

### Metabolic engineering for efficient supply of acetyl-CoA from fatty acid

Fatty acid is catabolized through the β-oxidation pathway into acetyl-CoA. Metabolic engineering for supplying acetyl-CoA from fatty acid was manipulated by derepression of *fadR* (a major regulator that negatively controls the fatty acid degradative regulon) and constitutive expression of *fadD* (a transporter involved in the transport and activation of exogenous fatty acid).

**Fig. 5:**
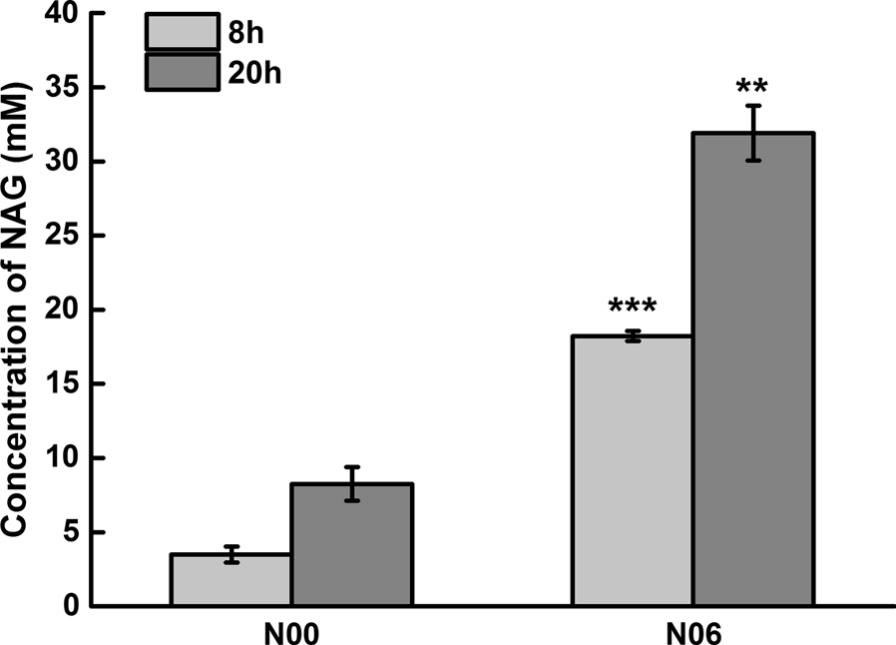
Production of NAG by engineered strain from glutamate and fatty acid. Concentrations of 50 mM sodium glutamate and 15 mM palmitic acid were used for NAG production. The bioconversion reactions were performed at 37 °C and 200 rpm

Host strain N06 was constructed by deletion of *fadR* and replacement of the native promoter of *fadD* with a strong promoter CPA1 in host N00. Strain 0619 with plasmid pN19 in host N06 used fatty acid efficiently and produced 31.92 mM NAG after 20 h of bioconversion with 50 mM sodium glutamate and 16 mM palmitic acid as substrates (Fig. 5). These results confirmed that an increased acetyl-CoA pool can be efficiently achieved from fatty acids through a modified β-oxidation pathway which increased NAG production by 3.8-fold.

### Comparison of three sources for acetyl-CoA supply

To compare the efficiency of different carbon sources for acetyl-CoA supply, 80 mM glucose, 160 mM sodium acetate, or 20 mM palmitic acid were used as substrate which produce mole equivalent of acetyl-CoA theoretically (Table 1). The carbon sources were added to the reaction system with 50 mM sodium glutamate for whole-cell bioconversion to achieve NAG production.

**Fig. 6:**
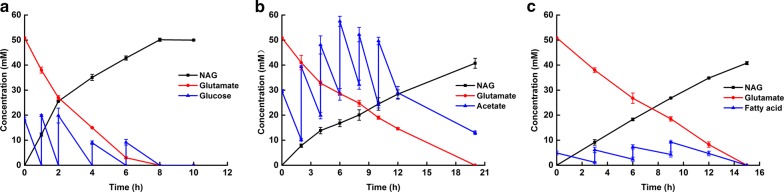
Comparison of glucose, acetate, and fatty acid as source of acetyl-CoA. The reaction mixture containing 50 mM sodium glutamate and 1 × M9 salts buffer were used. **a** Glucose (80 mM) was supplemented at rates of 20, 20, 20, 10, and 10 mM at 0, 1, 2, 4, and 6 h of bioconversion. **b** Acetate (160 mM) was supplemented at rates of 30, 30, 30, 30, 20, and 20 mM at 0, 2, 4, 6, 8, and 10 h of bioconversion. Additionally, 2 mM glucose was added at 0 h of bioconversion to supply ATP for the acetylation of acetate. **c** Palmitic acid (20 mM) was supplemented at 5 mM at each addition at 0, 3, 6, and 9 h of bioconversion

When glucose was used as an acetyl-CoA source, all 50 mM glutamate was transformed to NAG after 8 h of conversion (Fig. 6a) by strain 0419 with 98.2% and 62.4% molar conversion ratios of NAG from glutamate and glucose, respectively. When using acetate to produce acetyl-CoA, the reaction rate was much slower than that when using glucose. After 20 h of bioconversion, 40.73 mM NAG was obtained from strain 0019Ack and 13 mM acetate remained (Fig. 6b). After 20 h, the molar conversion ratios of NAG from glutamate and acetate were 81.4% and 27.7%, respectively. When using palmitic acid, a fatty acid, as the source of acetyl-CoA, 40.80 mM NAG was obtained by strain 0619 after 15 h of bioconversion. The titer of NAG was equal to that using acetate as a source by 0019Ack (Fig. 6c), but the productivity was higher. The molar conversion ratios of NAG from glutamate and palmitic acid were 81.6% and 204%, respectively.

The results showed that the strain engineered to use glucose (strain 0419) exhibited the highest conversion rate of glutamate and the highest productivity (an average of 6.25 mmol/L/h), indicating that the highest productivity of acetyl-CoA was achieved when glucose was used as a carbon source. The productivity of acetyl-CoA from acetate and fatty acid was not as efficient as that from glucose.

### Bioconversion for NAG production by engineered strain 0419

**Fig. 7:**
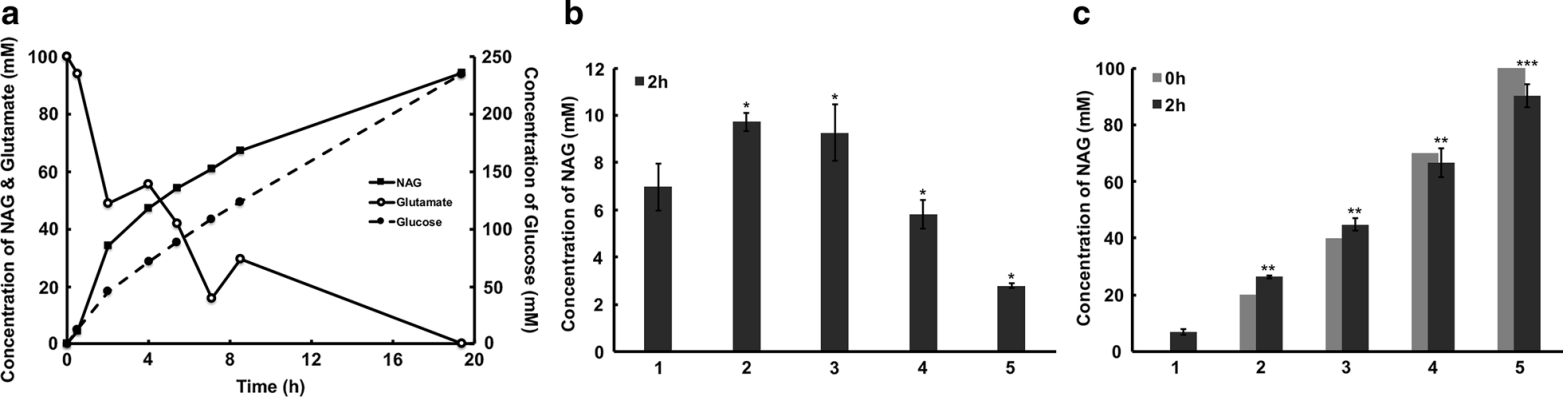
Bioconversion for NAG by engineered strain 0419. **a** Scale-up bioconversion of NAG production in a 1-L fermenter. The black square indicates the concentration of NAG. The hollow circle represents the concentration of glutamate. The black circle represents the concentration of glucose consumed. **b** Substrate inhibition. The concentrations of glutamate used were: 1: 50 mM; 2: 100 mM; 3: 200 mM; 4: 500 mM; 5: 1 M. The glucose concentration was 50 mM. The reaction was carried out for 2 h. **c** Product inhibition. The concentration of NAG at 0 h represented the initially added concentration. In detail, the concentrations of NAG added initially were: 1: 0 mM; 2: 20 mM; 3: 40 mM; 4: 70 mM; 5: 100 mM. The bioconversion reactions were performed at 37 °C and 200 rpm for 2 h

Strain 0419 produced NAG with a high yield and conversion rate using glucose as a carbon source for acetyl-CoA supply. Thus, scale-up bioconversion using strain 0419 was carried out in a 1-L fermenter containing 500 mL medium for NAG production. The substrate sodium glutamate was added at 100 mM in one portion, and the substrate glucose was added in a fed-batch manner. After 23 h of bioconversion, 94 mM NAG was obtained, while 230 mM glucose was consumed (Fig. 7a). The molar conversion rate of NAG from glucose was much lower in the fermenter than in the shaking flasks. This may be because acetyl-CoA was consumed in the tricarboxylic acid (TCA) cycle. The scale-up bioconversion process was also carried out with higher concentrations of glutamate (200 or 500 mM) added once or multiple times, but the NAG yield did not increase. The results indicated that there might be substrate inhibition or product inhibition in this reaction.

As shown in Fig. 7b, as glutamate levels increased, NAG production did not increase accordingly. When the concentration of glutamate was increased to 200 mM or more, the NAG yield decreased. These results suggest that glutamate inhibited NAG production. NAG production was reduced when the amount of NAG added initially was more than 40 mM. When the initial NAG concentration was more than 70 mM, NAG degradation was faster than NAG production (Fig. 7c). These results confirmed that a high concentration NAG inhibited NAG synthesis. The effects of NAG and glutamate on the Ks-NAGS activity were also analyzed in vitro, and the specific activity of Ks-NAGS reduced when the concentration of glutamate was higher than 20 mM and the concentration of NAG was higher than 0.5 mM (Additional file 1: Figure S1). The result was consistent with the in vivo experiment. The above results suggested inhibition of both substrate inhibition and product inhibition of NAGS from *Kitasatospora setae*. This limited the increase in NAG production during scale-up bioconversion.

## Discussion

Acetyl-CoA is the precursor of many high-cost chemicals and can provide an acetyl-group precursor for producing various industrially useful acetyl-metabolites. Metabolic engineering of *E. coli* for efficiently supplying acetyl-CoA would enhance the production of acetyl-chemicals. In this study, the biosynthesis of NAG, which is synthesized from glutamate and acetyl-CoA, was used to develop an efficient strategy for supplying acetyl-CoA. We first constructed a NAG-producing *E. coli* strain 0019 by knocking out native *argB* and *argA* and overexpressing a new NAGS from *K. setae*. However, Ks-NAGS was inhibited by both its substrate glutamate and its product NAG. Screening of NAGS with higher enzyme activity or the evolution of NAGS from *K. setae* may further improve the NAG production.

The NAG producing strain 0019 was further optimized by metabolic engineering using different carbon sources, including glucose, acetate, and fatty acid. Glucose was more efficient than acetate and fatty acid for supplying acetyl-CoA with the highest NAG productivity, but the yield of NAG from glucose remained far below the theoretical conversion rate. The active TCA cycle, which can consume large amounts of acetyl-CoA, may explain this discrepancy. Further studies to balance the acetyl-CoA flux to NAG and TCA cycle are needed to increase the yield of acetyl-CoA from glucose.

Acetate is a promising alternative carbon source for supplying acetyl-CoA in complex reactions because of its short pathway. Acetate has been used as a carbon source in the production of polyhydroxyalkanoates, itaconic acid, and other chemicals. Chen et al. used acetate as a main carbon source to synthesize polyhydroxyalkanoates, and strains overexpressing the ACK-PTA pathway produced nearly fourfold levels of poly-3-hydroxybutyrate, twofold levels of poly(3-hydroxybutyrate-*co*-4-hydroxybutyrate), and twofold levels of poly(3-hydroxybutyrate-*co*-3-hydroxyvalerate) compared to control strains [14]. By overexpressing *acs* and inactivating *iclR*, the conversion rate of acetate to itaconic acid was improved to 16.7% of the theoretical maximum yield [12]. In this study, a 27.7% conversion rate of acetate to NAG was obtained by overexpression of the ACK-PTA pathway and NAGS from *K. setae*. However, the reaction rate when using acetate as a carbon source was very slow, and the conversion rate remained far below the theoretical conversion rate. Further studies are needed to evaluate the assimilation of acetate, ATP supply, and TCA consumption for efficient conversion of acetate to acetyl-CoA.

Fatty acid is advantageous for synthesizing compounds such as *n*-butanol and 3-hydroxypropionate in processes that require a high reducing power. Liu et al. [29] developed an engineered *E. coli* strain that can efficiently use fatty acid as feedstock and produced 3-hydroxypropionate with a yield of 1.56 g/g FAs in a 5-L bioreactor. In this study, fatty acid was used to supply acetyl-CoA for NAG synthesis with an acceptable titer. The results indicated that fatty acid can also be used to provide an acetyl group for producing value-added acetyl-chemicals. Except for palmitic acid, other types of fatty acids can be tested in further studies, and more targets of the β-oxidation pathway should be identified to further improve the assimilation and utilization efficiency of fatty acid.

## Conclusions

In this study, systemic metabolic engineering of *E. coli* strains for efficiently supplying acetyl-CoA from glucose, acetate, and fatty acid was carried out and applied in the biosynthesis of NAG. Deletion of the native *argB* and *argA* and introduction of an efficient NAGS from *K. setae* resulted in an engineered *E. coli* strain for NAG production. Metabolic engineering strategies were then applied in the *E. coli* strain using different carbon sources to supply the precursor acetyl-CoA. The recombinant strains 0419, 0019Ack, and 0619 were used to compare acetyl-CoA supplied from 80 mM glucose, 160 mM acetate, and 20 mM palmitic acid for NAG production, respectively. The molar weights of glucose, acetate, and palmitic acid used theoretically produced 160 mM acetyl-CoA. For NAG synthesis, glucose was the most efficient carbon source, but acetate and fatty acid also showed good performance. The results demonstrated the great potential of acetate and fatty acid for supplying acetyl-CoA. The strategies developed in this study may be potentially applicable for producing not only acetyl-CoA-derived metabolites, but also a wide range of useful compounds that require acetylation in the pathway. However, further engineering is required to enhance the utilization of acetate and fatty acid by *E. coli* strains.

## Methods

### Strains and plasmids

The bacteria strains and plasmids used in this study are listed in Table 3. *Escherichia coli* DH5α was used for molecular cloning. *Escherichia coli* K12 (BW25113) was used as the parental strain for genetic modification and NAG production. Gene knockout strains were obtained from the KEIO collection (National BioResource Project) [30]. The plasmids pRB1 s and pSB1a used for gene expression were derived from expression vectors previously developed in our laboratory (unpublished) and have the following features: a promoter (araBAD), multiple cloning sites, rrnB terminator, origin of replication RSF1030 and pSC101, and streptomycin and ampicillin resistance genes. The nucleotide sequence of genes encoding enzymes that can catalyze NAG formation were obtained by PCR amplification and ligated into pRB1 s at the *Nco*I and *Eco*RI sites by the Gibson assembly method [31]. The templates for NAG synthase genes from *T. thermophilus*, *M. ruber*, *K. setae*, and *D. deserti* were synthesized and codon-optimized by Generay (Shanghai, China). Because NAGS from *E. coli* and *P. aeruginosa* have both an acetyl transferase domain and amino acid kinase domain [22], only the C-terminal proteins of *E. coli* ArgA and *P. aeruginosa* ArgA were amplified and cloned into pRB1 s. The plasmids pAcs and pAck were constructed by introducing the acs and ackA-pta operons of *E. coli* into the *Nco*I and *Eco*RI sites of pSB1a. For chromosomal phenotype integration, P1 virus-mediated transfection was performed as described by Thomason et al. [32]. The plasmid pCP20 was used to remove the FLP recognition target-flanked resistant marker in *E. coli* K12 [33]. For gene and promoter replacement, the CRISPR-Cas9 system was used [34]. The primers used in this study are listed in Additional file 1: Table S1.

**Table 3:** Strains and plasmids used in this study

Strain/plasmid	Description	Reference
Strains		
*E. coli* DH5α	*F*^−^*endA1 glnV44 thi*^−^*1 recA1 relA1 gyrA96 deoR nupG* Φ*80dlacZ* ∆*M15* ∆*(lacZYA*-*argF)U169, hsdR17(r*_*K*_^−^ *m*_*K*_^+^*), λ*^−^	Invitrogen
*E. coli* BW25113	*lacI*^*q*^*rrnB*_*T14*_∆*lacZ*_*WJ16*_*hsdR514*∆*araBAD*_*AH33*_∆*rhaBAD*_*LD78*_	Invitrogen
N00	*E. coli* BW25113, ∆*argB,* ∆*argA*	This study
N01	*E. coli* BW25113, ∆*argB,* ∆*argA*, ∆*ptsG::glk,* ∆*galR::zglf*	This study
N02	*E. coli* BW25113, ∆*argB,* ∆*argA*, ∆*ptsG::glk,* ∆*galR::zglf*, ∆*poxB::acs*	This study
N03	*E. coli* BW25113, ∆*argB,* ∆*argA*, ∆*ptsG::glk,* ∆*galR::zglf*, ∆*poxB::acs*, ∆*ldhA*	This study
N04	*E. coli* BW25113, ∆*argB,* ∆*argA*, ∆*ptsG::glk,* ∆*galR::zglf*, ∆*poxB::acs*, ∆*ldhA*, ∆*pta*	This study
N05	*E. coli* BW25113, ∆*argB,* ∆*argA*, ∆*ptsG::glk,* ∆*galR::zglf*, ∆*poxB::acs*, ∆*ldhA*, ∆*gltA*	This study
N06	*E. coli* BW25113, ∆*argB,* ∆*argA*, ∆*fadR, P*_*fadD*_*::P*_*CPA1*_	This study
Plasmids		
pRB1 s	RSF1030 origin, Pbad promoter, Str^R^	Our lab
pSB1a	pSC101 origin, Pbad promoter, Amp^R^	Our lab
pN01	*argA* from *E. coli* cloned into pRB1 s	This study
pN02	*argA* from *Pseudomonas aeruginose* cloned into pRB1 s	This study
pN03	*argA* from *Xanthomonas campestris* cloned into pRB1 s	This study
pN05	*argA* from *Corynebacterium glutamicum* cloned into pRB1 s	This study
pN06	*argA* from *Streptomyces coelicolor* cloned into pRB1 s	This study
pN08	*argA* from *Mycobacterium tuberculosis* cloned into pRB1 s	This study
pN14	*argA* from *Thermus thermophilus* cloned into pRB1 s	This study
pN18	*argA* from *Meiothermus ruber* cloned into pRB1 s	This study
pN19	*argA* from *Kitasatospora setae* cloned into pRB1 s	This study
pN20	*argA* from *Deinococcus deserti* cloned into pRB1 s	This study
pAcs	*acs* from *E. coli* cloned into pSB1a	This study
pAck	*ackA*-*pta* from *E. coli* cloned into pSB1a	This study

### Culture conditions

Luria-Bertani (LB) medium (per liter: tryptone 10 g, yeast extract 5 g, NaCl 10 g) was used for all molecular construction experiments and strain cultures. Strains harboring the temperature-sensitive plasmid pCP20 were incubated at 30 °C and 220 rpm, while the other strains were grown at 37 °C and 220 rpm. For protein expression, the strains were pre-cultured in LB medium supplemented with appropriate antibiotics (streptomycin 50 mg/L, ampicillin 100 mg/L) at 37 °C for 4–6 h, and then transferred into auto-inducing ZYM medium (per liter: tryptone 10 g, yeast extract 5 g, glycerol 5 g, glucose 0.5 g, l-arabinose 2 g, Na_2_HPO_4_ 25 mM, KH_2_PO_4_ 25 mM, NH_4_Cl 50 mM, Na_2_SO_4_ 5 mM, MgSO_4_ 2 mM and trace elements containing 0.05 mM FeCl_3_, 0.02 mM CaCl_2_, 0.01 mM MnCl_2_, 0.01 mM ZnSO_4_, and 0.002 mM each of CoCl_2_, NiCl_2_, Na_2_Mo_4_, Na_2_SeO_3_, and H_3_BO_3_) [35] with 1% inoculum and induced at 37 °C for 12–16 h.

### Whole-cell biocatalysis conditions

Whole-cell biocatalysis was used for NAG production [36]. Cells induced overnight were harvested by centrifugation at 4000 rpm for 10 min and washed with 0.85% NaCl solution once. The cells were suspended in 1 mL bioconversion medium containing 50 mM sodium glutamate and 1 × M9 salts (per liter: 12.8 g Na_2_HPO_4_.7H_2_O, 3 g KH_2_PO_4_, 0.5 g NaCl, 1 g NH_4_Cl) and different carbon sources to form a cell suspension from a starting biomass of OD_600nm_ = 30. The bioconversion reaction was performed at 37 °C and 200 rpm in a flask. In experiments comparing the three sources of acetyl-CoA, a reaction mixture containing 50 mM sodium glutamate and 1 × M9 salts was used. The substrate glucose, acetate, or fatty acid was supplemented frequently in small amounts based on the experimental conditions.

### Scale-up of bioconversion for NAG production using glucose as a substrate

The scale-up of bioconversion was carried out in a 1-L fermenter containing 500 mL bioconversion medium. Cells were induced first in a 5-L fermenter containing 2 L auto-inducing ZYM medium similar to that in the shake flasks, and then the cells were harvested by centrifugation. The induced cells were suspended in bioconversion medium containing 100 mM glutamate and 1 × M9 salts with an initial cell density of OD_600nm_ = 30. The cells were cultured at 37 °C with 30% dissolved oxygen. The pH was maintained at 7.0 by feeding of sodium hydroxide. Glucose was fed at an initial rate of 25 mM/h and slowed after 2 h when it accumulated. The feeding rate was then controlled to ensure accumulation of no more than 1 g/L.

### Enzymatic analysis of NAGS

NAGS activity was assayed spectrophotometrically by measuring the increase in absorbance at 412 nm due to the formation of 5-thio-2-nitrobenzoate resulting from the reaction between the sulfhydryl group of CoASH, generated by the amino acid acetylating activity, and 5,5-dithio-bis(2-nitrobenzoic acid) (DTNB) as previously reported [37]. Reaction mixtures contained 50 mM Tris–HCl (pH 8.0), 2 mM AcCoA, 20 mM sodium glutamate, 10 mM MgCl_2_, 0.2 mM DTNB and 0.65 μg enzyme in a total volume of 300 μL. Color production was linear with time for > 1 min, One unit of enzyme activity is defined as the amount of enzyme required to produce 1 μmol of NAG per minute at 37 °C.

### Substrate and product inhibition

For substrate inhibition analysis, 5 groups of bioconversion media were used containing 50 mM glucose and different concentrations of glutamate (50, 100, 200, and 500 mM and 1 M). Cells after culture were harvested and suspended in 1 mL conversion medium, and the reaction was carried out for 2 h at 37 °C and 200 rpm.

The product inhibition experiment was carried out by adding different concentrations of NAG (0, 20, 40, 70 and 100 mM) to the conversion medium containing 50 mM glutamate and 50 mM glucose. After conducting the reaction at 37 °C and 200 rpm for 2 h, NAG in the supernatants was detected.

### Analytical methods

Cell density was estimated by measuring the optical density at 600 nm. The concentrations of NAG, glucose, and organic acids in the supernatant were determined by high-performance liquid chromatography equipped with a Bio-Rad Aminex HPX-87H Ion Exclusion column (7.8 × 300 mm; Hercules, CA, USA), refractive index detector, and diode-array detector. Analysis was performed at 65 °C with a mobile phase of 5 mM H_2_SO_4_ at a flow rate of 0.5 mL/min.

## Additional file

**Additional file 1: Figure S1.** The effects of NAG and glutamate on Ks-NAGS activity. **Table S1** Primers used in this study.

## Data Availability

Not applicable.

## Abbreviations

NAG: *N*-acetlyglutamate
NAGS: *N*-acetlyglutamate synthase
Ec: *E. coli*
Pa: *Pseudomonas aeruginose*
Xc: *Xanthomonas campestris*
Cg: *Corynebacterium glutamicum*
Sc: *Streptomyces coelicolor*
Mt: *Mycobacterium tuberculosis*
Tt: *Thermus thermophiles*
Mr: *Meiothermus ruber*
Ks: *Kitasatospora setae*
Dd: *Deinococcus deserti*
TCA: tricarboxylic acid
LB: Luria–Bertani
