# Supplementary material for: Metabolic engineering for efficient supply of acetyl-CoA from different carbon sources in Escherichia coli
Source: Microb Cell Fact. 2019 Aug 6;18:130. doi: 10.1186/s12934-019-1177-y (PMC6685171; doi:10.1186/s12934-019-1177-y)
Supplement: Supplementary file 1 — Additional file 1: Figure S1. The effects of NAG and glutamate on Ks-NAGS activity. Table S1 Primers used in this study. [file 12934_2019_1177_MOESM1_ESM.docx]

**Additional file**

**Figure S1** The effects of NAG and glutamate on Ks-NAGS activity.

| 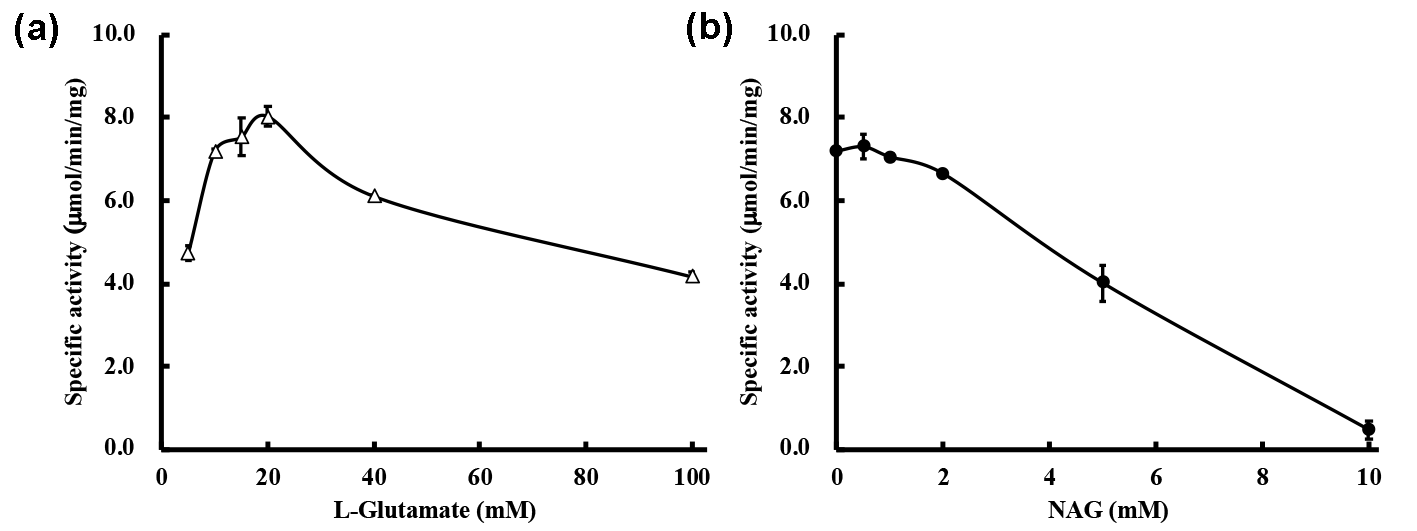 |
| --- |

**Table S1** Primers used in this study.

| Primers | Sequence (5'-3') |
| --- | --- |
| Ec-NcoI-F | gctaacaggaggaattaaccatggaaagcgccgagcagat |
| Ec-EcoRI-R | gctgcagaccgagctcaccgaattcttaccctaaatccgccatca |
| Pa-NcoI-F | gctaacaggaggaattaaccatgcaggagcagttcgagca |
| Pa-EcoRI-R | gctgcagaccgagctcaccgaattctcacaggctcttctcgaata |
| Xc-NcoI-F | gctaacaggaggaattaaccatggagtcgtcggtgtcgatc |
| Xc-EcoRI-R | gctgcagaccgagctcaccgaattctcaccccagcaaggtgggtt |
| Cg-NcoI-F | gctaacaggaggaattaaccatgacgcctagtcttccccg |
| Cg-EcoRI-R | gctgcagaccgagctcaccgaattcttagaatttccgttcggcgt |
| Sc-NcoI-F | gctaacaggaggaattaaccatgtcaaatgccatcagcgt |
| Sc-EcoRI-R | gctgcagaccgagctcaccgaattctcacagatgcagaagcatcc |
| Mt-NcoI-F | gctaacaggaggaattaaccatgaccgaacgtccacggga |
| Mt-EcoRI-R | gctgcagaccgagctcaccgaattcctacagcaccagcagcatcc |
| Tt-NcoI-F | gctaacaggaggaattaaccatgcgtggtctgtctctgtc |
| Tt-EcoRI-R | gctgcagaccgagctcaccgaattcttagccaccgctcgcacccg |
| Mr-NcoI-F | gctaacaggaggaattaaccatgagcaccatcgaaattgg |
| Mr-EcoRI-R | gctgcagaccgagctcaccgaattcttaggcgcccggtttgcgat |
| Ks-NcoI-F | gctaacaggaggaattaaccatggaagtgaccattcgtcg |
| Ks-EcoRI-R | gctgcagaccgagctcaccgaattcttacagatgcagcagcatac |
| Dd-NcoI-F | gctaacaggaggaattaaccatgaccttcctggcactgga |
| Dd-EcoRI-R | gctgcagaccgagctcaccgaattcttattcgagggctttaaacatg |
| acs-NcoI-F | ggctaacaggaggaattaaccatgagccaaattcacaaaca |
| acs-EcoRI-R | gctgcagaccgagctcaccgaattcttacgatggcatcgcgata |
| ackA-NcoI-F | ggctaacaggaggaattaaccatgtcgagtaagttagtactg |
| pta-EcoRI-R | gctgcagaccgagctcaccgaattcttactgctgctgtgcagact |
